# Citrullination Post-Translational Modification: State of the Art of Brain Tumor Investigations and Future Perspectives

**DOI:** 10.3390/diagnostics13182872

**Published:** 2023-09-07

**Authors:** Diana Valeria Rossetti, Alexandra Muntiu, Luca Massimi, Gianpiero Tamburrini, Claudia Desiderio

**Affiliations:** 1 Istituto di Scienze e Tecnologie Chimiche “Giulio Natta”, Consiglio Nazionale delle Ricerche, 00168 Rome, Italy; dianaross79@hotmail.com; 2 Dipartimento di Scienze Biotecnologiche di Base, Cliniche Intensivologiche e Perioperatorie, Università Cattolica del Sacro Cuore, 00168 Rome, Italy; alexandra.muntiu@gmail.com; 3 UOC Neurochirurgia Infantile, Dipartimento di Scienze dell’Invecchiamento, Neurologiche, Ortopediche e della Testa-Collo, Fondazione Policlinico Universitario A. Gemelli—IRCCS, Università Cattolica del Sacro Cuore, 00168 Roma, Italy; luca.massimi@unicatt.it (L.M.); gianpiero.tamburrini@unicatt.it (G.T.)

**Keywords:** post-translational modifications, citrullination, deimination, brain tumors

## Abstract

The present review aims to describe the state of the art of research studies investigating the citrullination post-translational modification in adult and pediatric brain tumors. After an introduction to the deimination reaction and its occurrence in proteins and polypeptide chains, the role of the citrullination post-translational modification in physiological as well as pathological states, including cancer, is summarized, and the recent literature and review papers on the topic are examined. A separate section deals with the specific focus of investigation of the citrullination post-translational modification in relation to brain tumors, examining the state of the art of the literature that mainly concerns adult and pediatric glioblastoma and posterior fossa pediatric tumors. We examined the literature on this emerging field of research, and we apologize in advance for any possible omission. Although only a few studies inspecting citrullination in brain tumors are currently available, the results interestingly highlighted different profiles of the citrullinome associated with different histotypes. The data outlined the importance of this post-translational modification in modulating cancer invasion and chemoresistance, influencing key factors involved in apoptosis, cancer cell communication through extracellular vesicle release, autophagy, and gene expression processes, which suggests the prospect of taking citrullination as a target of cancer treatment or as a source of potential diagnostic and prognostic biomarkers for potential clinical applications in the future.

## 1. Introduction: Protein Citrullination Post-Translational Modification

Post-translational modifications (PTMs) of proteins play an important role inside and outside of cells in physiological as well as pathological processes. PTMs can involve the covalent addition of chemical groups and/or proteolytic cleavage at amino acid residues positioned along the sequence or at the N- and C-protein termini [1]. Protein PTMs influence the folding, function, and degradation of a protein and therefore have an important role in the regulation of biological processes [2]. Currently, increasing attention is focused on characterizing PTMs and mapping their alterations in pathological conditions, with the aim of identifying specific biological markers or investigating the molecular pathways involved in the onset of diseases. The identification of PTMs is, however, an analytical challenge because they largely occur in sub-stoichiometric amounts, and specific methods of enrichment for their proteomic analysis and characterization are often necessary [3].

**Figure 1:**
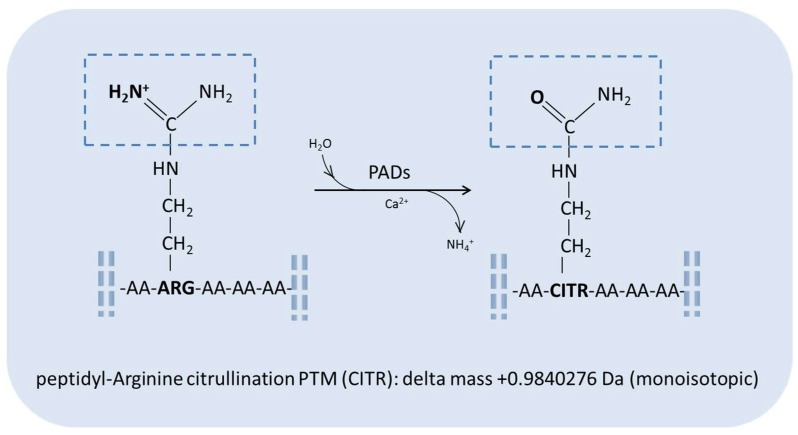
General scheme of the peptidylarginine citrullination reaction.

The citrullination PTM, specifically involving arginine amino acid residues, has been associated with physiological processes and various diseases, such as rheumatoid arthritis, lupus, multiple sclerosis, Alzheimer’s disease, and different forms of cancer [4,5,6,7]. This PTM consists in the conversion of peptidylarginine in peptidylcitrulline in a calcium-dependent reaction catalyzed by peptidylarginine deiminases (PADs), a family of tissue-specific enzymes, i.e., PAD 1–4 and PAD 6 isoenzymes [6,8]. This reaction results in the deimination of arginine with the substitution of a keto group, leading to the formation of citrulline and the release of ammonia (Figure 1). The modification generates a molecular mass increase of 0.9840276 Da (monoisotopic) in the peptide/protein chain and the loss of a positive charge, changing the physico-chemical properties as well as the conformation, function, and interaction of the protein/peptide carrying the modification, including possible denaturation, especially when PTM dysregulation occurs [9,10]. In fact, the strong basic character of arginine amino acid residues, which can be easily protonated at a physiological pH, is lost when citrulline is formed, with the latter containing, conversely, neutral urea in its side chain. This modification influences the chromatographic behavior of the modified protein/peptide, which shows, together with the molecular mass increase, a slight shift in its retention time with respect to the unchanged form due to the stronger interaction with reverse-phase chromatographic columns, favoring its distinguished detection and characterization by high-resolution mass spectrometry detectors.

Peptidylarginine citrullination by PAD enzymes is dependent on the position of the amino acid along the sequence. About 80–90% of citrullinated arginine residues are positioned after an aspartic acid residue. This PTM barely occurs at the N-terminal sequence or close to glutamic acid residues [6,8,11]. A recent proteomic study mapped protein citrullination in 30 human tissues via mass spectrometry characterization, database searching, and validation of 375 citrullination sites on 209 human proteins, enlarging the knowledge on the proteins carrying this modification and on the preferred sites of citrullination around Asp and Gly amino acid residues [5].

PAD isoenzymes are Ca^2+^-dependent enzymes that have tissue-specific expressions, with several cytoplasmatic, membrane, nucleic, and mitochondrial proteins as targets [11,12]. In particular, PAD1 is mainly expressed in the epidermis and uterus, and it deiminates intermediate filaments, keratin, and filaggrin, with physiological roles in skin differentiation. PAD2 is localized in various tissues, including the central nervous system (CNS), glial cells, the uterus, skeletal muscle, the pancreas, secretory glands, inflammatory cells, and mitochondria, and targets several proteins including myelin basic protein, vimentin, and glial fibrillary acidic protein. PAD3 is expressed in keratinocytes and hair follicles, while PAD4 is expressed in granulocytes and different cancer cell types and is localized in the cytoplasm and nucleus. PAD4 substrates comprise several proteins, including vimentin, histones, nucleophosmin, and p300. PAD6 has been detected in eggs, ovaries, and embryos and lacks catalytic activity due to mutations. PAD4 was also found to citrullinate methylarginine in addition to arginine through demethylimination reactions in histones [13].

PAD enzymes have a different number of sites of calcium binding (from four to six), and the binding of calcium generates a conformational change in the enzyme with the activation of its catalytic site [4,9]. As outlined by Zhang et al. [14], PADs also have an affinity for barium divalent ions; nevertheless, their calcium specificity and affinity are higher, with PAD activation occurring at supraphysiological Ca^2+^ concentrations. Therefore, under physiological conditions, PAD enzymes are generally inactive unless dysregulated calcium homeostasis conditions occur, such as in epidermal differentiation and cell death (apoptosis or necrosis), or in altered bicarbonate concentrations or redox states [9,15]. The half-maximal activity of PADs can be achieved at 40–60 μM calcium concentrations, and it has to be taken into account that calcium has a different concentration inside and outside of cells. Intracellular calcium concentrations (cytosolic) are in the range of 10^−5^–10^−3^ mM, while extracellularly they reach concentrations of 1.1–1.3 mM (plasma concentration) [15]. It is, however, established that citrullination plays a role in physiological processes that increase calcium concentrations, which, in turn, activate PAD enzymes or produce calcium variations through a mechanism that has yet to be fully clarified [6,9]. Different review papers recently described the citrullination PTM, providing an overview of its role in physiological as well as pathological processes [4,5,6,7,16] and demonstrating the increasing attention that this PTM has captured in recent years. In addition, the diagnostic and therapeutic potentials of the citrullination PTM and/or PAD enzyme activity modulation were also specifically discussed in relation to different diseases, including cancer [4,14,16]. 

The present review aims to illustrate the state of the art of research studies investigating the protein citrullination PTM, specifically in brain cancer, which, to the best of our knowledge, has yet to be described, and outlines potential diagnostic perspectives. We examined the literature on this emerging field of research, and we apologize in advance for any possible omission.

## 2. Citrullination PTM in Physio-Pathological Processes

Although widely studied in relation to different pathological states, protein citrullination has important roles in physiology because it regulates several processes involving alterations in calcium concentrations, such as skin cell differentiation, epidermal barrier maintenance, embryo development, gene regulation, CNS plasticity, apoptosis, and immune responses [4,5,6,7,16]. Citrullination can induce protein unfolding, thus favoring protein degradation, or, conversely, it can hamper protein degradation, favoring polymerization, making the modified protein an important element in the regulation of the different physio-pathological processes that stimulated the study of this PTM in cells and tissues for the characterization of the so-defined “citrullinome” [16,17,18].

PAD activation in physiological processes can be linked to calcium level variations as well as other mechanisms, such as the binding of enzymes to proteins or a cofactor that causes a conformational change that reproduces the calcium switch, or activation by phosphorylation, autocitrullination, or components of the EGF signaling pathway [16]. 

The increase in calcium occurring during epidermis terminal differentiation activates PAD enzymes that have a role in skin, hair, and nail thickness and consistency, as well as in preserving the moisture of the cutaneous barrier, through the citrullination of structural proteins, such as suprabasal cytokeratins K1 and K10, profilaggrin, and vimentin in epidermal cells [6,18,19,20]. During differentiation, the structural protein trichohyalin (THH), which is expressed in hair follicles, is citrullinated by PAD3 following increases in calcium concentrations. In this form, it becomes the substrate of transglutaminases and stabilizes the keratin matrix reticular structure [6,18]. A novel role of the citrullination PTM in elastogenesis was also recently outlined [21]. 

In apoptotic cells, citrullination plays a role in regulating the assembly–disassembly of vimentin filaments, the disorganization of the nuclear lamina, and protein-unfolding-mediated nucleosome collapse following the activation of PAD enzymes by increased calcium concentrations [6]. The importance of PAD enzymes in apoptosis was evident following the observation of apoptosis and adhesion interruption in PAD2-knockout macrophages as a result of caspase activation and phosphokinase downregulation [22]. Vimentin expression was found on the cell surface of PAD2-overexpressing cells and activated Jurkat cells while they were undergoing apoptosis [23]. PAD3 was demonstrated to have a role in the homeostasis of human neural stem cells through the regulation of calcium-induced and caspase 3-independent cell death by cytoskeleton disassembly and altered nucleus translocation of apoptosis-inducing factor (AIF) [24]. 

Citrullination has an important role in CNS plasticity in children, with myelin basic protein (MBP) involved as a PAD substrate. Citrullination reduces the charge of the MBP protein, lowering the interaction with negatively charged phospholipids and influencing myelin organization. The protein is fully citrullinated up to 2 years of age, while the modified form decreases to approximately 18% after 4 years of age and in adults [6,25].

PAD-mediated citrullination has a regulatory role in the formation of neutrophil extracellular traps (NETs), an extracellular defense mechanism of the organism against microbial infections through the assembly of structures formed by nuclear proteins and decondensed chromatin released from cells in the extracellular environment. In case of infections, the activated neutrophils stimulate phagocytosis and other mechanisms of defense including the formation of NETs (NETosis), which act on bacteria extracellularly [7]. NETosis is regulated by PAD4 in peripheral blood neutrophils through histone citrullination and unfolding, which are necessary to produce chromatin decondensation and the formation of NETs [26]. The role of PAD4 in NETosis was demonstrated by the decrease in the formation of NETs in PAD4-deficient mice [10]. PAD4 and PAD2 play a role in NET formation through the citrullination of histone 3 [18,27]. Other immune cells, such as macrophages, mast cells, and eosinophils, can generate NETosis [28], outlining the role of histone citrullination in other processes involving NETosis. Therefore, histones are established substrates of PAD enzymes, and citrullination of histones plays an important role in NETosis and other processes such as gene regulation. Indeed, PAD4 is the isoform of the PAD family of enzymes, which includes a nuclear transfer signal, thus allowing this enzyme to participate in gene regulation [20]. 

PAD4 has been shown to convert both arginine and methylarginine residues in histones H3 and H4 to citrulline, resulting in demethylimination and methylamine release in the latter case [13]. Thus, gene regulation via PAD citrullination of histones also involves histone demethylation, with histone methylation representing an on/off signal of gene expression undergoing epigenetic modulation. Histones’ citrullination occurs in both health and disease conditions, underlining the importance of this specific histone modification in several pathophysiological processes [10]. Gene regulation by PAD4 can also involve the interaction of the enzyme with non-histone proteins, such as the tumor suppressor p53; in this case, protein citrullination in apoptosis and cell growth processes is controlled in a p53/PAD4-dependent way [10]. A mutual effect in gene regulation has also been found between PAD4 and estrogens. Guo and Fast [10] identified the tumor suppressor protein Inhibitor of Growth 4 (ING4) as a PAD4 substrate. PAD4 citrullinates the p53 binding domain of the protein, inhibiting ING4/p53 interactions [29]. In addition, citrullination can affect the action of chemokines and cytokines through modification by PAD4 and PAD2 enzymes of specific substrates which alter their function and potency or modulate pathways directly or indirectly involved in the control of their gene transcription and epigenetic regulation, with important roles in inflammatory-based diseases and cancer [30]. 

The abnormal activity of PAD enzymes can have pathological effects due to hyper- or hypo-proteins’ citrullination, with consequences in the alteration of the related physiological processes and molecular signaling. Citrullination has been shown to be implicated in various pathological processes, with several studies focused on rheumatoid arthritis (RA), a chronic autoimmune disease characterized by the infiltration of activated macrophages and inflammatory synovium [6,7,9,20]. RA exhibits high concentrations of autoantibodies against numerous targets and, in particular, of anti-citrullinated protein antibodies (ACPAs). ACPAs are highly specific for this disease, and enzymatic assays using cyclic citrullinated peptides (CCPs) are routinely used in RA diagnosis [6,7]. In the synovium of RA patients, the leucocyte infiltrate releases PAD2 and PAD4, producing large amounts of citrullinated proteins, including fibrinogen, vimentin, collagen type II, and alpha-enolase, which, in turn, activate the immune response through the generation of anti-citrulline antibodies against these modified protein antigens [31]. 

Méchin et al. examined citrullination in skin diseases such as psoriasis, a disease characterized by skin lesions of unknown etiology, probably due to abnormal keratinocyte hyperproliferation and differentiation and infiltration of inflammatory cells into the dermis and epidermis. Immunohistochemistry revealed a lack of citrullinated keratin K1 in skin specimens of psoriatic lesions compared to normal epidermis [32]. A reduced quantity of citrullinated K1 has also been reported in bullous congenital ichthyosiform erythroderma (BCIE) [32].

The control of citrullination is essential in the CNS; in fact, alterations in this protein modification have been recognized in neurodegeneration and multiple sclerosis (MS), an autoimmune disease in which the degradation of the myelin sheath of neurons, synthesized by oligodendroglial cells, causes disturbances in electrical conduction [6,9]. MS is associated with the hypercitrullination of MBP, with the modified form of the protein reaching 45%, and sometimes up to 90% in severe forms, compared with the physiological percentage that drops to around 18% in children >4 years and adults [33,34]. Hence, in MS, the citrullinated/uncitrullinated MBP ratio changes and replaces the ontogenetically initial state [6]. Citrullination triggers the partial unfolding of MBP and enables the protein to be more easily degraded by proteases, such as cathepsin D [6,7,34].

An abnormal amount of protein citrullination has been found in reactive astrocytes from prion disease brains [16], and in the hippocampus and extracellular amyloid plaques from postmortem Alzheimer’s disease (AD) brains [16,35]. Vimentin and glial fibrillary acidic protein (GFAP) are included in the list of the citrullinated proteins of the hippocampus [6,32]. Both diseases exhibit dysregulated calcium levels that can stimulate protein citrullination in the CNS [6,9]; nevertheless, the mechanism by which the deregulation of PAD may promote diseases is not yet clear [16].

A recent study investigating protein citrullination in amyotrophic lateral sclerosis (ALS) in two different model mice demonstrated changes in protein citrullination in association with disease progression. Notably, protein citrullination increased in astrocytes and decreased in neurons during disease progression. The enrichment of citrullination in protein aggregates suggested a role of this PTM in the generation of the myelin protein aggregates in ALS disease, which contribute to myelin degeneration [36]. 

A proteomic investigation performed specifically on the citrullination PTM revealed about 150 proteins to be substrates of the PAD4 enzyme, of which about 25% contained the RG/RGG motif in the sequence, which therefore proved to be a consensus sequence for citrullination by PAD enzymes. Citrullination by PAD4 was also revealed to inhibit the methylation of the RGG motif in the FUS, EWS, and TAF15 FET proteins and hnRNPA1, as well as their aggregation, involved in ALS pathogenesis [37]. 

Recently, the citrullination PTM has been identified in matrix metalloproteinases in neutrophil-rich sputum samples from patients affected by cystic fibrosis [38].

In cancer, protein citrullination may have a significant role in the pathogenesis as well as the progression of the disease [10,14,39,40,41,42,43]. In the tumor microenvironment, the citrullination PTM has a role in tumor cell signaling by acting as an on/off switch of different modes of transcriptional regulation [39,40,41,42,43]. In recent papers, the citrullination of histones and other proteins, such as alpha-enolase, 60 kDa heat shock protein mitochondrial, keratin cytoskeleton chain 8 type II, and beta tubulin, has been shown to facilitate the development of cancer through different mechanisms [41,42]. Two members of the family of PAD enzymes, PAD2 and PAD4, have been found to be expressed at high levels in tumor tissues and are considered useful diagnostic markers and possible therapeutic targets [10,43]. In particular, in adenocarcinoma, PAD4 overexpression showed co-location with cytokeratins 8, 18, and 19, which are common tumor markers. The finding of citrullinated forms of cytokeratins, characterized by a recognized resistance to digestion by caspases, defines a potential role of PAD4 in modulating this mechanism of apoptosis in tumor cells [39]. 

PAD4 levels were found to be elevated in blood from patients with malignant tumors, but not in specimens from patients affected by benign tumors [40].

PAD2 has been shown to mediate histone citrullination in various types of cancer, such as prolactinoma, multiple myeloma (PAD2-CitH3R6), breast cancer MCF-7 (PAD2-CitH3R2, 8, 17), and prostate cancer (PAD2-CitH3R26), where, particularly in prostate cancer, its expression is required for the survival and cell cycle development of prostate cancer cells [10]. While a low expression of PAD2 and PAD4 is correlated with a low survival rate in primary CRC tumors, the levels of PAD4 and citrullination in the extracellular matrix have been reported to be important for the processes of adhesion and migration of colon cancer cells contributing to the progression of liver metastasis [44]. PAD4-driven citrullination affects numerous biological processes such as the epithelial–mesenchymal transition (EMT), proliferation, metastasis, apoptosis, and DNA damage [10,14,41]. Its high expression can be found in metastases and malignant tumors, and approximately 40% of cells in malignant lymphomas express PAD4 [40]; in contrast, no enzyme expression has been observed in benign tumors and non-tumor tissues, with the exception of some inflamed tissues [40,41]. The citrullination of histones by PAD4 has been detected in various types of cancers, such as acute myeloid leukemia (PAD4-H3), lung cancer (PAD4-CitH4R3), non-small cell lung cancer (PAD4-CitH4R3), gastric cancer (PAD4-CitH3R26), osteosarcoma (PAD4-CitH3) [10], and, like PAD2, prolactinoma (PAD4-CitH3R2, 8, 17) and breast cancer MCF-7 (PAD4-CitH3R17) [10,41]. PAD4-mediated citrullination of histones in tumors, also favored by stressful conditions such as surgery, hypoxia, high levels of fatty acids, and DNA damage, leads to the decondensation of chromatin, increasing the risk of tumor progression through NETosis, as previously described [10,14,45]. Specifically, in glioma malignant tumor cells, surgical stress followed by hypoxia can lead to the formation of NETs and citrullinated proteins [46], conditions predicting poor postoperative survival among cancer patients [10]. The molecular mechanism of NETosis promotes tumor proliferation, growth, invasion, and distant metastasis by degrading the extracellular matrix, trapping the circulating tumor cells using adhesive substrates, awakening dormant tumor cells (causing cancer recurrence), damaging vascular integrity, inducing stromal endothelial transformation, and promoting angiogenesis [10,14]. Histone-citrullination-mediated NETs occur at the aggregation site of tumor neutrophils, and their accumulation has been observed in renal cell carcinoma, melanoma, glioblastoma, hepatocellular carcinoma, colorectal carcinoma, gastric carcinoma, esophageal carcinoma, lung cancer, ovarian cancer, and head and neck cancer [10]. As recently reported, tumor migration and metastasis can be modulated by PAD4-mediated citrullination through another molecular mechanism involving the formation of extracellular chromatin networks (CECNs) [14], which are chromatin DNA network-like structures, similar to NETs, released into the extracellular space of cancer cells [47]. 

## 3. Citrullination as Target of Potential Treatments in Cancer

The pharmacological inhibition of PADs may be an effective application to reduce tumor cell proliferation without affecting the viability of normal cells [41]. Currently, several reversible inhibitors of PAD enzymes have been identified, such as paclitaxel, minocycline, and streptomycin; however, they exhibit relatively weak action [10]. Several irreversible inhibitors, including Cl-amidine, instead show much greater efficacy [41]. An alternative potential application could be to detect the titer of anti-citrullinated peptide antibodies in cancer patients [41]. Hypoxia-generated necrosis in malignant glioma cells could activate PAD enzymes, resulting in extracellular protein citrullination, for example, in body fluids, where the calcium concentration is suitable for PAD activation, opening up potential clinical applications in the future. Moreover, the citrullinated peptide epitopes present in tumor cells [48] could be used as targets for vaccination and cancer therapy, or as tumor neoantigens to stimulate the immune response and to induce antitumor immunity [14,41,48]. 

The following paragraph specifically illustrates the investigation of the citrullination PTM in relation to brain tumors.

## 4. Citrullination PTM and Brain Tumors: State of the Art and Directions

A recent paper that mapped the citrullination PTM in thirty human tissues [5] described finding the largest number of modified proteins in brain tissue. This is not surprising given the above-described role of the citrullination PTM in the physiological development of the CNS in childhood and neurodegenerative diseases. Although the citrullination PTM has been extensively studied in relation to neurodegeneration, a limited number of articles deal with specific investigations in relation to adults and pediatric brain tumors. 

PAD expression was investigated in U-251MG cells of human astrocytoma under normal and dibutyryl cAMP-supplemented cell cultures [49]. PAD2 and PAD3 expression was found to be mediated by cyclic adenosine monophosphate-protein kinase A (cAMP-PKA) signaling; however, the study did not involve the characterization of citrullinated proteins. 

Based on previous findings of the hypoxia-induced expression of PAD2 and citrullinated glial fibrillary acidic protein (GFAP) in astrocytes and that of PAD4 in different tissues and biofluids of malignant tumors [39,40], Sase et al. studied the expression of PAD enzymes and protein citrullination in U-251 MG human cells of malignant glioma under normoxia and hypoxia via quantitative polymerase chain reaction (qPCR) and two-dimensional electrophoresis (2-DE) mass spectrometry proteomics [46]. PAD enzyme isoforms 1, 2, 3, and 4 were found to be significantly overexpressed under hypoxic conditions, and this effect was found to be dependent on the hypoxia-inducible factor (HIF-1), the main regulator of the cellular response to hypoxia. Nevertheless, it was found that the citrullination of intracellular proteins occurred in cells only after lysis and exposure to calcium, therefore following processes activated ex vivo. However, more consistent protein citrullination was found in cell lysates cultured under hypoxia that involved cytoskeletal, stress-related, and glycolytic proteins, such as vimentin (VIME), GFAP, filamin-A (FLNA), 78 kDa glucose-regulated protein (GRP78), actin cytoplasmic 1 or 2 (ACTB or ACTG), glyceraldehyde-3-phosphate dehydrogenase (G3P), and alpha-crystallin B chain (CRYAB). In the same study, PAD2 was shown to citrullinate in vitro the vascular endothelial growth factor receptor VEGFR-2, responsible for the generation of autoantibodies in RA. Therefore, it cannot be excluded that VEGFR2 citrullination may also generate antigens in high-grade malignant glioma, a possibility to be further investigated [46]. 

Glioblastoma multiforme, the most aggressive adult brain tumor, has been the target of other studies involving protein citrullination in an attempt to elucidate the molecular mechanisms underlying its high recurrence rate and ability to infiltrate distal brain sites, aimed at investigating the role of PAD-mediated pathways in the release of extracellular vesicles (EVs) by cancer cells. Indeed, increased expression of PADs has been correlated with an increased release of EVs, which have a role in tumors by contributing to disease progression and tumor chemoresistance. Inhibitors of PAD enzymes have been shown to have synergistic effects in both reducing EV release from cancer cells and inhibiting exosomes, as well as in microvesicle-mediated tumor chemoresistance [50,51].

LN299 chemosensitive and LN18 chemoresistant GBM cell lines have been reported to exhibit different levels of PAD2–4 enzymes. In particular, LN299 cells showed higher levels of PAD3, while LN18 cells showed higher levels of PAD2 and PAD4. Proteomic analysis after immunoprecipitation showed the citrullination of mitochondrial and cytoskeletal proteins and proteins associated with stress and invadopodia, including prohibitin, a mitochondrial protein involved in chemoresistance, and histone H3. Several processes were found to be affected in GBM cells following the inhibition of pan-PAD enzymes by Cl-amidine, namely, a consistent reduction in EV release and modulation of the microRNA composition, favoring anti-oncogenic components over pro-oncogenic ones, as well as a validated reduction in prohibitin and histone H3 citrullination, both in single and combined treatments with temoxolodime [50]. However, cell viability was not affected by the administration of Cl-amidine in GBM cells. The citrullinated forms of prohibitin and histone H3 have been reported to have specific roles in cancer, especially in apoptosis, cell survival, and EV release. By comparing the two cell lines, LN18 chemoresistant GBM cells showed higher levels of the prohibitin protein, which were reduced, considering both the unmodified and deiminated forms, when a pan-PAD inhibitor was added to the culture. Other proteins involved in the progression, cell invasion, and migration of cancers, including GBM, have been found carrying the citrullination PTM in both the cell lines studied, namely, AHNAK, STIM1, moesin, cathepsin D, GAPDH, histones, and histone deacetylase (HDAC). Annexin-1 and Integrin beta-1 were found to be citrullinated only in LN229 chemosensitive GBM cells [52]. These data demonstrate the involvement of PAD enzyme activity, and thus protein citrullination, in the modulation of GBM cell communication, signaling, and chemoresistance, suggesting that treatment of GBM with PAD inhibitors against specific targets represents a potential therapeutic strategy. 

The role of protein deimination and PAD enzymes in the modulation of EVs’ release and their molecular composition in the CNS was recently reviewed, with a focus on GBM among the CNS tumors examined [53,54]. In one review article, the perspectives of pan-PAD enzyme inhibitors and/or citrullinated protein signature applications in clinics for the early diagnosis and treatment of CNS diseases were also outlined. The different citrullinome profiles in male and female GBM patients revealed an interesting correlation with data from our recent top-down proteomic investigation of pediatric brain tumor tissues of different grades of aggressiveness and location, namely, medulloblastoma, pilocytic astrocytoma, ependymoma, and GBM [55]. Citrullination was identified in numerous peptide fragments of GFAP and vimentin naturally occurring in the intact proteome, and, in particular, in their C-terminal peptide fragments, which exhibited one or more PTM sites. Deiminated arginine was frequently contained in the sequence traits KTVETRDG and KTVEMRDG of the vimentin and GFAP proteins, respectively. In accordance with the loss of the positive charge of arginine through deimination, the modified form of the peptide showed a different chromatographic behavior to that of the unmodified form, resulting in different retention times under the operating condition used; the higher the retention time, the greater the number of citrullinated arginine residues. Furthermore, in mono-citrullinated peptides, different sites of the PTM inside the sequence differently influenced the physico-chemical properties of the peptide and therefore its chromatographic retention time. The presence of vimentin and GFAP peptide fragments together, as well as their citrullinated forms, mainly characterized tumor tissues of lower World Health Organization (WHO) grades, such as pilocytic astrocytoma and ependymoma, while they were negligible in the most aggressive medulloblastoma. In pediatric GBM, according to WHO grade IV of this tumor, few GFAP fragments were detected, and vimentin fragments, when present, showed a different distribution between male and female patients, highlighting sexual dimorphism in pediatric GBM disease. Like the unmodified forms, the citrullinated peptide fragment of GFAP and vimentin therefore appeared to be associated with pediatric brain tumors with a lower degree of aggressiveness, according to the WHO classification. Wang et al. reviewed the roles of PAD2- and PAD4-mediated protein citrullination in various forms of cancers and its controversial function in promoting tumor development or, conversely, lowering its malignancy, depending on the tumor location and pathway involved [56]. Vimentin and GFAP citrullination is produced by the PAD2 enzyme [23,35], and the identification of citrullinated peptides of these proteins in less aggressive histotypes of pediatric brain tumors would suggest a potential role in clinical prognostic applications. It is also noteworthy that citrullinated vimentin peptides with the same sequence as the citrullinated vimentin peptides found in pediatric brain tumor tissues [55] have been described for their immunoregulatory and anti-inflammatory properties in rheumatoid arthritis [57].

## 5. Conclusions and Future Perspectives

Protein post-translational modifications play an important role in both physiological and pathological states, and their aberration is implicated in the events that trigger disease onset or disease progression through the structure and function of proteins and the associated intra- and extra-cellular processes. Post-translational modifications have recently been highlighted for their potential role in clinical applications, particularly in cancer [58]. In this regard, citrullination could therefore be included for its potential clinical applications in the diagnosis as well as treatment of cancer, and for its roles in the modulation of apoptosis and EV release from tumor cells, and cancer chemoresistance. Indeed, protein citrullination by PAD enzymes has been reported to play a role in cancer development and progression by modulating several processes, such as the epithelial–mesenchymal transition (EMT), tumor progression and metastasis, apoptosis, gene regulation, and autophagy [10,14,41], resulting in an intriguing target for the development of new treatments and the identification of diagnostic/prognostic biomarkers in tumor tissues and liquid biopsies. 

The importance of citrullination in cancer diagnosis was recently outlined in a review of the state of the art of PTM research in colorectal cancer (CRC) [44]. Citrullination and PAD activity marked CRC, but PAD and citrullinated proteins may have different or even opposite roles. Additionally, the citrullination of histone H3, catalyzed by PAD4 and associated with NETs, can play a role in the epithelial–mesenchymal transition of CRC cells, stimulating progression and metastasis. Willumsen et al. measured the levels of various peptides and proteins, including citrullinated vimentin, in the serum of patients with lung, gastrointestinal, and prostate cancers, malignant melanoma, chronic obstructive pulmonary disease, and idiopathic pulmonary fibrosis and compared them with those of age-matched control samples. The levels of citrullinated vimentin were found to be significantly higher in lung cancer than in controls and the other tumors studied [59]. The elevated levels of the citrullinated protein in all stages of the disease suggest the use of citrullinated vimentin as an early disease biomarker. 

The detection of anti-citrullinated protein antibodies (ACPAs) in serum for the diagnosis of rheumatoid arthritis [60,61] has increased interest in studying citrullinated proteins in other minimally invasive biofluids, such as saliva [62]. From this point of view, saliva could represent a useful biofluid to deeply investigate the rate of protein citrullination in adult and pediatric brain tumors and to discover potential biomarkers of diagnosis and early recurrence. This perspective is supported by a recent review paper that interestingly outlines the role of conventional and unconventional secreted proteins/peptides in brain tumor cell communications and microenvironments, and in tumor survival and progression [63], considering that secreted proteins include several substrates of PAD enzymes. A recent paper outlined a role of protein citrullination in the Warburg effect, which occurs in cancer cells, hyper-stimulating the glycolysis process through the regulation of PKM2 by deimination [64], which should also be investigated in brain tumors, since, to the best of our knowledge, it has not yet been studied. 

Although brain tumors are rare tumors, they represent the most common solid tumors in pediatric age; a full understanding of the molecular mechanisms underlying their development and progression is still required. The different profiles of tumor tissue citrullinomes suggest that further investigation into the role of citrullination in different brain tumor histotypes is needed. Furthermore, single-cell proteomics, together with increasingly sophisticated bioinformatics tools, is currently a high-demand tool for characterizing tumor cell heterogeneity and mapping the proteome and its PTMs, including citrullination, to better decipher the immunosuppressive hallmark of tumors, such as the role of tumor-associated macrophages and migroglia (TAMs) in glioblastoma [65], however, taking into account that the identification of PTMs is always an analytical challenge [3].

Although few studies have investigated the role of protein citrullination and/or PAD enzymes specifically in brain tumors, the literature data stimulate further investigations that could reveal distinguished biomarkers or novel therapeutic molecular targets involving specific deiminated proteins/peptides. For this purpose, integrated genomic and proteomic platforms represent essential tools for matching gene expression data with the complexity of the protein phenotype and its post-translational modifications, such as PAD expression and protein-related deimination. 

## Data Availability

The literature data illustrated in this review article can be retrieved from the relevant cited references detailed in the reference list.
